# Endoglucanases: insights into thermostability for biofuel applications

**DOI:** 10.1186/1754-6834-6-136

**Published:** 2013-09-27

**Authors:** Ragothaman M Yennamalli, Andrew J Rader, Adam J Kenny, Jeffrey D Wolt, Taner Z Sen

**Affiliations:** 1 Department of Genetics, Development and Cell Biology, Iowa State University, Ames 50011, IA, USA; 2 Department of Physics, Indiana University-Purdue University Indianapolis, Indianapolis 46202, IN, USA; 3 Biosafety Institute for Genetically Modified Agricultural Products and Department of Agronomy, Iowa State University, Ames 50011, IA, USA; 4 Bioinformatics and Computational Biology Program, Iowa State University, Ames 50011, IA, USA; 5 Present Address: Department of Biochemistry and Cell Biology, Rice University, Houston, TX 77005, USA; 6 Present Address: State Farm Insurance, Indianapolis 46240, IN, USA; 7 Present Address: Brownells, Inc, Montezuma, IA 50171, USA; 8 1025 Crop Genome Informatics Lab, Iowa State University, Ames 50011, IA, USA

**Keywords:** Biofuel, Endoglucanase, Thermostability, Cellulases, Mutant

## Abstract

Obtaining bioethanol from cellulosic biomass involves numerous steps, among which the enzymatic conversion of the polymer to individual sugar units has been a main focus of the biotechnology industry. Among the cellulases that break down the polymeric cellulose are endoglucanases that act synergistically for subsequent hydrolytic reactions. The endoglucanases that have garnered relatively more attention are those that can withstand high temperatures, i.e., are thermostable. Although our understanding of thermostability in endoglucanases is incomplete, some molecular features that are responsible for increased thermostability have been recently identified. This review focuses on the investigations of endoglucanases and their implications for biofuel applications.

## Introduction

The use of plants as bioreactors is not a new concept. Within the last fifteen years, studies have established that with the knowledge of biotechnology and genetic engineering, plants are indeed a low-cost source to produce stable molecules: plants are harnessed to produce antibodies, biodegradable plastics, recombinant proteins, carbohydrates, and fatty acids. A major goal of plant-based bioreactor technology is the production of stable enzymes to produce industrially useful products. Among these enzymes are manganese-dependent lignin peroxidase (for bleaching of pulp), phytase (for animal feed), (1–3,1-4)-β-glucanase (for brewing), and xylanase (for animal feed, paper and baking), which are used in processing plants such as alfalfa, tobacco, and barley [1-8]. In addition, as a result of growing environmental concerns of consuming fossil-based fuels, enzymes used in the production of plant-based ethanol (i.e., bioethanol) gained more importance in recent years, including α-amylases and endoglucanases.

α-amylase and endoglucanase are both involved in the conversion of plant material into sugar; however, there is a critical difference when it comes to which part of the plant they catalyze: while α-amylases break down starch from maize grain, which is primarily used as food by humans and animals, endoglucanases break down cell-wall cellulose primarily from maize stover (i.e., leaves and stalk), which has been historically considered as waste. Despite the obvious drawbacks of using a crucial food item as a fuel source, maize grain remains a preferred choice as the biofeedstock for the ethanol production, because of its ease of harvesting in a large acreage and of introducing new traits.

The demand for bioethanol is expected to increase significantly in the near future: according to the Billion Ton Study published in August 2011 [9], 76 million tons of maize is used for the production of ethanol and yields 14.2 billion gallons of ethanol fuel per year. By 2017, the consumption of biomass for biofuel production is projected to be 103 million tons. Therefore, with the ever-increasing demand for energy sources, there is a strong need to look at other non-grain sources of biomass. While many feedstock sources (e.g., perennials such as switchgrass and Miscanthus, and biomass from forests) may take several years to become fully developed as resources, maize stover is a readily available resource for biofuel production [9].

Some efforts have been made to reduce the cost of cellulose breakdown in maize stover to make the process more cost-effective, and therefore more attractive for the bioethanol industry. Traditionally, industrial enzymes are added into a batch of reagents in reactors during chemical processing. Controlling pH and temperature levels become critical, because even small environmental variations can cause enzyme denaturation and subsequent loss of enzymatic activity [10]. Cellulose-based bioethanol processing therefore requires several reactors, including one for a high-temperature pre-treatment (~75°C) and another for low-temperature endoglucanase-mediated cellulose conversion into sugar. One strategy currently being explored to improve the efficiency and lower the cost of these conversion steps is to develop feedstocks (such as maize stover) with heterologously expressed thermostable endoglucanases. Such a strategy will allow bypassing the need to add an externally sourced enzyme and possibly reduce number of processing steps [11-14]. To help in these efforts, we need to understand what factors, including protein sequence, structure, and dynamics, can be used to engineer thermostable endoglucanases.

Thermostability is a complex property that can be controlled by several factors, which may be working additively. Many studies comparing mesostable and thermostable proteins concluded that following properties may increase thermostability in general: selective pressure of certain amino acids [15], increase in hydrophobicity [16], change in a single amino acid [17], increase in compactness [18], increase in positively charged amino acids [19], and Gibbs free energy change of hydration [20]. These studies are usually general in the sense that they compare proteins across protein families, folds, sizes, and cellular location. When it comes to a specific protein family, however, our knowledge is usually limited as to what differentiates thermostable and mesostable proteins in that protein family.

In this review, we will first provide a short description of endoglucanase-mediated cellulose conversion process. Then we will return our attention to the main question of the thermostability-sequence-structure relationship in endoglucanases.

### Breaking down cellulose

Although the composition varies between different plants, 40-55% of the plant biomass is comprised of cellulose (a homopolymer made of repeating units of glucose), 25-50% of hemicelluloses (a heteropolymer made of glucose and other sugars) [21], and the remaining 10-40% is made up of lignin (a complex organic polymer) [22]. Conversion of cellulose polymers to simple sugars requires the use of cellulases. Cellulase is comprised of three distinct classes of enzymes (endoglucanases, cellobiohydrolases, and β-glucosidases) that act synergistically to break down the polymer. Endoglucanases act by cleaving internal β-glycosidic bonds in the cellulose chain, thereby making chain ends accessible to cellobiohydrolase. The end product cellobiose is further broken down to glucose units by β-glucosidase. Endoglucanases have an enzyme classification number 3.2.1.4 and belong to the broader enzyme group called glycosyl hydrolases, which also includes other cellulases such as exoglucanase and β-glucosidase. According to the CAZy database (http://www.cazy.org), endoglucanases are part of 13 distinct glycosyl hydrolase families, distributed in several archeal, bacterial, fungal, and eukaryotic organisms.

### Cellulose to biofuel conversion

From the agricultural field to the gas station, biofuel production from cellulosic biomass involves numerous steps that are strategically designed for efficient conversion [23]. In one of the earlier steps, pre-processing of the biomass occurs at high temperatures (75°C) coupled with a dilute sulfuric acid treatment [24]. This is done to liberate lignin, hemicellulose, and other compounds, and make the cellulosic polymers available for enzymatic degradation. Inefficient liberation of these compounds can lead to the enzyme inhibition, and therefore, reduce the efficiency of cellulose conversion. Currently, the efficiency of ethanol production from lingocellulosic biomass is physically and economically constrained by the pretreatment processes and the subsequent addition of cellulases to affect cellulose breakdown [25-27].

Industrial cellulases, particularly endoglucanases, are currently obtained from the fermentation of fungal and bacterial sources that are added to the production batch after the pretreatment step. The expression of endoglucanases from these external sources adds to the final cost of the bioethanol product. There is an intense interest in exploiting the potential of thermostable bioprocessing enzymes [28,29].

Due to the pH and temperature extremes involved in the biomass-to-biofuel conversion, stable endoglucanases and its synergistic enzymes, such as cellobiohydrolase and β-glucosidase, are sought for the enzymatic conversion of biomass. Thermostable endoglucanases from extremophiles are considered promising because they typically exhibit valuable characteristics in biofuel production including optimal functionality at higher temperatures and the ability to withstand extreme pH changes [30].

The strategy of heterologously expressing a thermostable endoglucanase in maize stover bypasses the need to add an externally sourced enzyme [11,14]. This way, the enzyme (in the biomass itself) can breakdown cellulose immediately following the pre-processing step. Furthermore, if the stable enzyme is expressed in the host tissue for biomass conversion, there can be substantive gains in production efficiency. This concept was shown to work previously with a non-cellulase enzyme during the breakdown of starch in maize grain: a synthetic chimera of three wild-type α-amylases from the archea *Thermococcales* that have an optimal growth temperature of above 80°C [31]*.* Introducing endoglucanase into plants reduces the recalcitrance of cellulose [12,13], since endoglucanases are capable of making random internal cleavage of the polymer, whose hydrolysis products are used by other enzymes. Transgenic plant expression is ideal for many reasons, including (a) almost unlimited scale-up potential, (b) cheap production – i.e. photosynthesis, (c) correct protein folding, (d) lack of human pathogens, and (e) the potential for direct use [32]. Cellulases currently account for approximately $0.68 to $1.47 per gallon of ethanol produced from cellulosic feedstocks [33], but this cost component could be reduced 5-fold with *in planta* expression [34-36].

A closer look at endoglucanases originating from bacterial sources shows significant diversity in their optimal temperature for enzymatic function. Some of these are optimally functional at elevated temperatures and thus thermostable. In the next section, we are interested in the following questions: What could be the differences between a thermostable and a mesostable endoglucanase? If there are any, can we translate these differences to modify an existing endoglucanase into a more thermostable protein? We will pursue the answers to these questions by reviewing computational analyses of thermostability in endoglucanases.

## Thermostability in endoglucanases

Enzymes that can (a) withstand high temperatures, (b) resist unfolding, and (c) perform their optimal activity at higher temperatures are called thermostable or thermophilic. Thermostable enzymes are highly stable at elevated temperatures where mesostable enzymes become denatured and thus lose their optimal activities [30,37,38].

Generally speaking, thermostability is a desired quality for proteins that have industrial and therapeutic significance [28,29,39-44]. Introducing thermostability has been one of the major focuses of protein engineering studies, specifically of computational studies, which can be divided in three broad categories in terms of number of enzymatic families and organisms analyzed: the proteome of a thermophilic organism to the proteome of a mesophilic organism [19][45], (b) proteins from multiple organisms belonging to a range of different protein families [15,16,18,20,46-48], and (c) a single protein family between thermophilic and mesophilic organisms [17,49,50].

Such studies have identified various factors imparting thermostability, including sequence-level factors (specific amino acids like Arg and Glu being significantly higher in thermophiles) and structure-level factors (energy of unfolding, number of Van der Waals contacts per residue, number of hydrogen bonds per residue, or number of residues involved in secondary structure). Other factors such as Gibbs free energy change of hydration, long-range non-bonded energy, and hydrophobicity, have also been mentioned. Although most of the studies attempted to identify “universal” factors imparting thermostability across protein families, at the protein family level, another set of factors (e.g., a different amino acid composition) may determine thermostability [51]. This inability to identify common features responsible for thermostability provided evidence for the view that no single rule defines thermostability [49].

In the previous studies of thermostable endoglucanases, Panasik et al. [49] analyzed the thermostability factors for proteins belonging to GH families that have the identical (α/β)_8_ fold. The lack of Gly in thermostable glycosyl hydrolase (which includes endoglucanase) was identified as the responsible factor. However, this analysis falls short in the following areas when it comes to its applicability to other endoglucanases: (a) the criteria of selecting the dataset of 29 proteins were based on higher crystallographic resolution rather than on diverse sequence identities, (b) only three endoglucanase structures were studied, and (c) the study does not individually analyze endoglucanases, but as part of a larger group of GH families.

Recently, a directed evolution approach has been used to identify an endoglucanase with higher thermostability from a thermophilic endoglucanase [52]. Although directed evolution has the ability to successfully find a mutant with higher stability and similar enzymatic activity, it is not guaranteed to do so. As such, finding a desirable mutant is a matter of trial and error, and does not necessarily explain why a particular mutant is thermostable or not. A promising method is using SCHEMA, where the structure of the protein is also used. This was used in developing thermostable chimeric cellobiohydrolases, of which many exhibited higher stability and optimal activity [53,54].

## Current computational thermostability studies with respect to Endoglucanases

### Protein structures tell a fold-dependent pattern of thermostability

Abundant structural information is present for endoglucanases in three different folds; namely (α/β)_8_, β-jelly roll, and (α/α)_6_ fold. Segregating and analyzing the structural data on the basis of these three different fold families has indicated that the type of fold is critical in identifying specific thermostabilizing features for endoglucanases [51].

**Figure 1:**
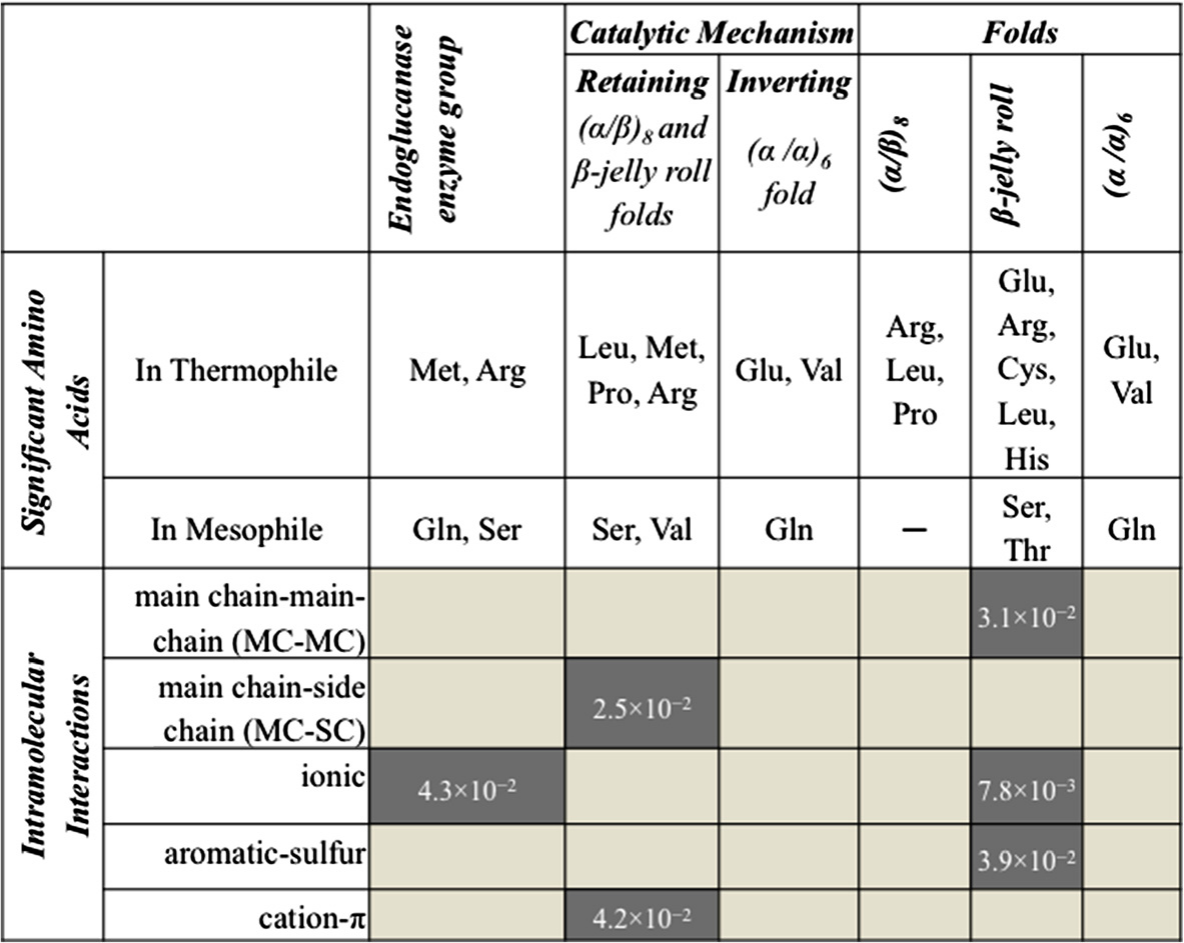
**Statistically significant amino acids and intramolecular interactions between thermostable and mesostable endoglucanases.** They are shown in thermostable endoglucanases as an enzyme group, based on the reaction mechanism, and within each fold, as compared to their mesostable counterparts (p-value < 5.0 × 10^-2^). The other interactions such as side chain-side chain interactions, hydrophobic interactions, disulphide bonds, and aromatic interactions were analyzed but found not to be statistically significant. The statistically significant intramolecular interactions for thermostable are shaded in dark gray. Note that the results for inverting mechanism and on (α/α)_6_ fold are the same. Figure reproduced with from a BioMed Central publication under the Creative Commons Attribution License from [51].

When comparing thermostable and mesostable endoglucanase enzymes as a whole, the amino acids Met and Arg and ionic interactions were significantly more enriched in thermophiles (See Figure 1). However, the enrichment of these amino acids is not statistically significant when considered at the fold-level analysis. In contrast, the solvent accessibility and secondary structure preference (placement in a helix, strand, or loop) showed a fold-dependent preference. Pairwise analysis of structurally similar endoglucanases (i.e. a pair of thermostable and mesostable endoglucanases) from the same fold showed distinct patterns of amino acid substitutions in the mesophile as compared to thermophile: in β-jelly roll fold, the amino acid Arg is replaced by Pro, and hydrophobic amino acids (such as Trp, Tyr, Phe, Iso, Met, Leu) indicating decreased ionic interactions in the mesostable endoglucanases.

### Structural dynamics also plays a role in thermostability

Proteins are constantly in motion, and an understanding of their dynamics will inform their functions [55]. Coarse-grained models, such as elastic network models, help in identifying the biologically meaningful motions of a protein [56]. Unlike molecular dynamics, which analyzes the dynamic motions of a protein at the atomistic level and are fine-grained, elastic network models perform at the amino acid level and are coarse-grained. An advantage of using coarse-grained models is the ability to observe the longer time-scaled motions in a short time-scale [56].

As a proof of concept, the dynamics of a pair of thermostable and mesostable endoglucanases using coarse-grained models was compared to identify dynamic differences [57]. For this purpose a pair of structurally highly similar endoglucanases with lower sequence similarity was selected. The ENM analyses showed that both the thermophile and mesophile displayed open/close and shear-type motions in the slow modes, and the loops that face the substrate binding side were observed to be more mobile than the non-substrate binding side. In contrast, the thermophile had large dynamic blocks moving in concert within the catalytic domain, providing more stability to the thermostable protein. Differences were also observed in the catalytic residues (i.e., nucleophile and acid/base donor): in thermophiles they showed positively correlated motions while they remain uncoupled in the mesophile.

### Single mutation and thermostability

Even a single mutation can significantly increase the thermostability of cellulases and their optimal activities [17,52,58]: (1) a Cys to Ser mutation of a cellobiohydrolase resulted in an increased thermostability by 8°C and a 10-fold increase in expression [58] and (2) two positions of an endoglucanase were identified to be crucial for increasing activity [52]. These studies provide evidence that only a few mutations can improve stability and activity significantly. Unfortunately two of these studies lack experimentally determined tertiary structures [52,58] and their claim of intramolecular bonding as a possible candidate for imparting thermostability is not easy to validate without a structure.

**Figure 2:**
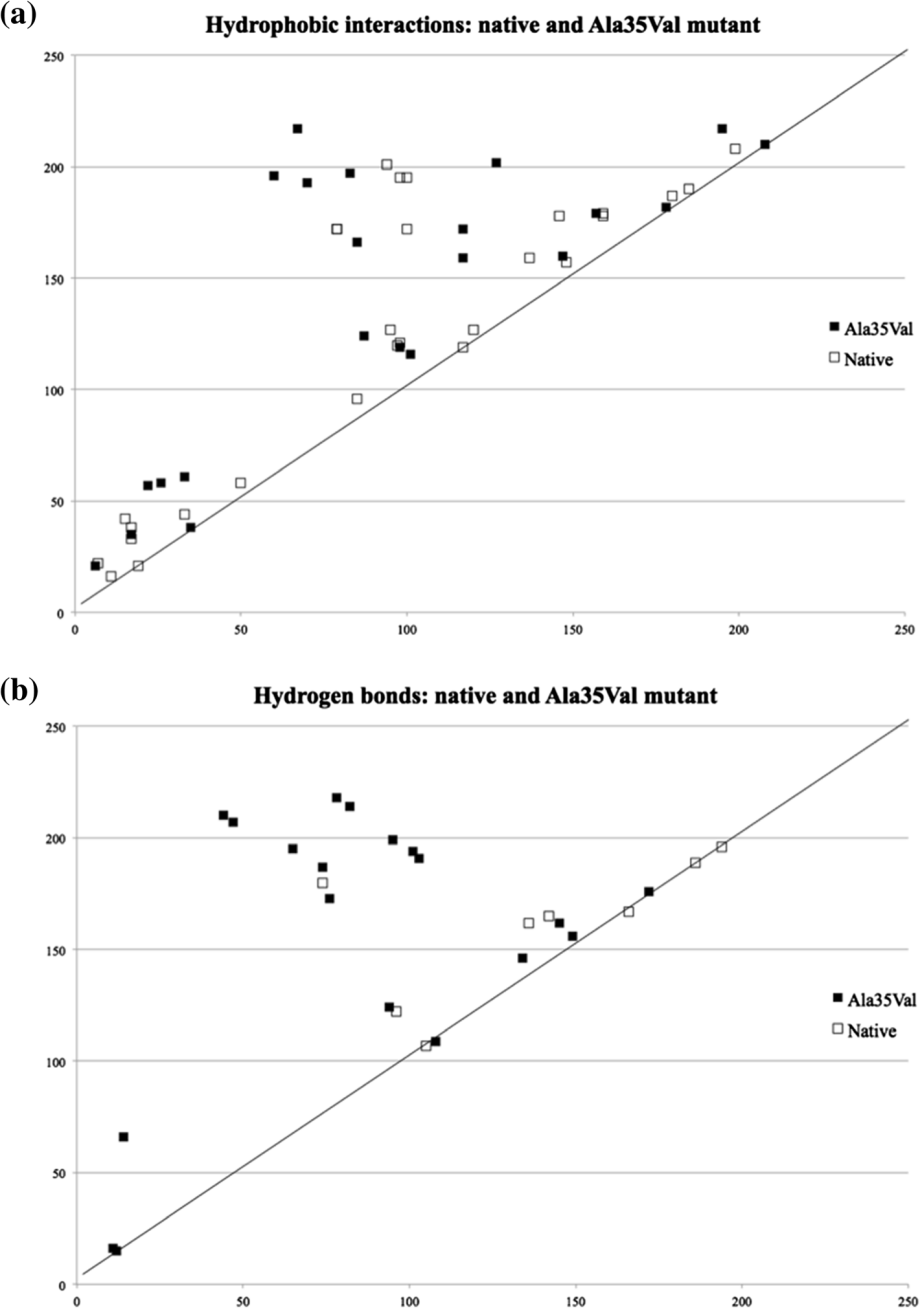
**Differences in contact maps between native and thermostable mutant (A35V) GH12 endoglucanase proteins.** Contacts present in the native but missing from the A35V mutant are in hollow squares. Similarly, contacts present in A35V but missing from native are in black squares. **(a)** Shows the hydrophobic interactions while **(b)** shows the hydrogen bonds at Θ = 346 K. Notice that the thermostable mutant in general has more contacts that are further off the slope = 1 diagonal leading to an increase in contact order (CO). This figure is reproduced from [57], published with permission from Taylor & Francis.

In general, mutating residues close to active sites are expected to enhance/disrupt the stability and function of a protein. In a puzzling case, as it is mentioned above, the Ala to Val mutation reported by Sandgren et al., the mutated residue is spatially distant from the active site (~20 Å) [17]. In order to study this puzzle further, two methods were used: FIRST [59] and contact order [60]. Using the software FIRST, a simulated thermal denaturation was performed and the temperature where the protein transitions from rigid to flexible (i.e., the theoretical melting temperature) was identified. Contact order (CO) provides a measure of the protein topology correlating with the folding rate of a given protein. After the CO value was calculated, it was observed that the newly formed hydrogen bonds and hydrophobic interactions were globally distributed in the protein (See Figure 2). This suggested that these new, long-range interactions have a stabilizing effect on the protein. It was also argued that a single distal mutation as Ala to Val at the 35^th^ position possibly acts as a trigger to create many other structural rearrangements without disturbing the flexibility of the active site in binding to the substrate [61]. Therefore, the effect of the single mutation is felt globally, rather than locally in the active site, imparting to the protein an overall increase in the thermostability.

## *Acidothermus cellulolyticus* E1: a promising enzyme

The E1 endoglucanase [pdb id: 1ece] from *Acidothermus cellulolyticus* is one of the promising enzymes for improving biofuel production efficiency. It has been expressed in prokaryotic systems for laboratory referencing [62,63] and has also been successfully expressed and quantified in many plant-based systems (Table 1) such as: (a) tobacco [12,64-68], (b) maize [12,36,69-71], (c) Arabidopsis [72], (d) potato [73], and (e) rice [74]. E1 has also been expressed in duckweed, an aquatic plant with a high starch content [75].

**Table 1:** List of plant species where E1 endoglucanase has been expressed and its activity studied

***Plant Species***	***Subcellular compartment***	***% E1 of TSP***	***Source***
Tobacco *(Nicotiana tabacum)*	chloroplast	0.18 - 1.35%	[65]
	cytosol	0.0007 - 0.015%	[64]
	chloroplast	0.003 - 0.67%	
	apoplast	0.09 - 1.6%	
	apoplast	2.5%	[67]
	apoplast	0.25%	[66]
	chloroplast	0.06 - 12.0%	[68]
Maize *(Zea mays)*	apoplast	2.10%	[69]
	apoplast	0.01 - 1.16%	[71]
	endoplasmic reticulum	0.2 - 2.0%	[36]
	mitochondria	0.1 - 0.2%	
	endoplasmic reticulum	2.00%	[70]
	apoplast	nd	[12]
Arabidopsis *(Arabadopsis thaliana)*	apoplast	1.0 - 26.0%	[72]
Potato *(Solanum tuberosum)*	chloroplast	0.73 - 2.6%	[65]
	apoplast	0.38 - 0.92%	
Duckweed *(Lemna minor)*	cytosol	0.24%	[75]
Rice *(Oryza sativa)*	apoplast	2.4 - 4.9%	[74]

Many studies have analyzed different aspects of E1 expression: the level of heterologous expression [65], maximum recovery of recombinant enzyme after expression [68], retention of enzymatic activity after the ammonia fiber explosion treatment (AFEX) process [67], ability of E1 to access cell wall components [12], stability of E1 in various sub cellular compartments [66], and maximum accumulation through specific subcellular targeting [64]. In maize tissues where E1 was expressed, studies focused on E1 stability after expression [69], activity on AFEX-treated stover [71], tissue specific production [36], synergistic action with other cellulases [35], and its effect on whole plant digestibility [12].

As of now, E1 expressed in plants has to be harvested and added exogenously to the ethanol process after pretreatment. In this respect, two studies focus on the feedback effect of pre-treatment processes on endoglucanase activity. Brunecky et al. reported that maize stover expressing E1 exhibited greater degradability due to E1 actively hydrolyzing the plant cell wall during growth [12]. This increased processability was particularly interesting as E1 was substantively active during plant growth at ambient temperatures, however, after pretreatment processes (up to 170°C heat) E1 was not considered active in subsequent saccharification. Similarly, Teymouri et al. found that E1 present in biofeedstocks lost at least 65% of the original activity after the ammonia fiber expansion (AFEX) pre-treatment process. AFEX is a pretreatment process that combines use of ammonia under moderate pressure (up to 400 psi) and high temperature (up to 200°C). This causes the cellulose fibers to separate from depolymerized lignin. It has been noted that AFEX is a mild pretreatment strategy compared to alternative pretreatment processes such as steam explosion or acid treatment [67]. Additionally, E1 has been expressed *in planta* (apoplast) and was found to have internal activity during plant growth that initiated the breakdown of plant cell walls before harvest [12]. This in turn would increase the processability of feedstocks during stover-to-ethanol conversion.

Therefore, more studies are required on E1 that relate to thermostability and E1’s synergistic action in an enzyme cocktail, expression of hyper-thermostable mutants of E1 and its localization, pretreatment technologies, and overall processability.

## Conclusion

Production of bioethanol from cellulosic biomass has come to a stage where newer catalysts are required for efficient production. In this review, we have discussed a specific endoglucanase (E1) that is currently seen as an important catalyst, and the factors contributing to thermostability that can be exploited for cellulosic bioethanol production. We explored the question of “*What parameters need to be considered to engineer an endoglucanase with suitable thermodynamics, stability, and higher activity?”* Any mutant or engineered endoglucanase that preserves the thermostability in terms of the dynamics is a potential industrial candidate. In reviewing literature and current structural studies, we observed that although “universal” rules that impart thermostability are useful for engineers, each protein family is different, and may have different factors imparting thermostability. In endoglucanases, protein sequence, structure, and dynamics all play a critical role in stabilizing the endoglucanase at high temperatures.

In the same vein, engineers should keep in mind the kinetic and thermodynamics constraints of other cellulolytic enzymes (cellobiohydrolase, ligninase, β-glucosidase and E1 endoglucanase) that act synergistically to break down the polymer, which we have not covered in this review [76]. Screening for specific residual activity of endoglucanases in the presence of multiple enzymes can also improve enzyme selection.

## Abbreviations

CAZy: Carbohydrate-active enZYmes; GH: Glycosyl hydrolases; ENM: Elastic network model; FIRST: Floopy inclusions and rigid substructure topography; CO: Contact order; E1: Endoglucanase from *acidothermus cellulolyticus*; AFEX: Ammonia fiber explosion treatment.

## Competing interests

Authors declare that there are no competing interests.

## Authors’ contributions

TZS, JDW, and RMY conceived the idea. RMY wrote the first draft of the manuscript. All authors contributed to manuscript revision and approved the final version.
